# Monotherapy Versus Combination Therapy of Janus Kinase Inhibitors With Conventional Synthetic Disease-Modifying Antirheumatic Drugs (csDMARDs) in Rheumatoid Arthritis: Evidence From a Systematic Review of Randomized Controlled Trials

**DOI:** 10.7759/cureus.94538

**Published:** 2025-10-14

**Authors:** Razaz Galal Mirghani Idris, Aliya Awad Mohammed Nogod, Eman Mohamedalamin, Fatima Mohammed Ahmed Elhaj, Awadelgeed Widatalla Yousif Musa, Mohammed Elzaki Mohammed Mansoor, Ehab A Elagab

**Affiliations:** 1 General Medicine, Sligo University Hospital, Sligo, IRL; 2 Rheumatology, Cambridge University Hospitals NHS Foundation Trust, Cambridge, GBR; 3 Internal Medicine, Arrowe Park Hospital, Wirral, GBR; 4 Internal Medicine, Gold Trust Medical Center, Dubai, ARE; 5 Acute Medicine, The Princess Alexandra Hospital NHS Trust, Harlow, GBR; 6 Internal Medicine, Wexford Hospital, Wexford, IRL; 7 Pathology, College of Medicine, Najran University, Najran, SAU

**Keywords:** combination therapy, csdmards, efficacy, jak inhibitors, janus kinase inhibitors, monotherapy, rheumatoid arthritis, safety, systematic review

## Abstract

The optimal use of Janus kinase inhibitors (JAKi) in rheumatoid arthritis (RA), either as monotherapy or in combination with conventional synthetic disease-modifying antirheumatic drugs (csDMARDs), remains a subject of clinical debate. While combination therapy is often standard, JAKi monotherapy presents a potential alternative for patients intolerant to csDMARDs. This systematic review aims to evaluate and compare the efficacy and safety of JAKi monotherapy versus combination therapy with csDMARDs in patients with RA.

This review was conducted in accordance with Preferred Reporting Items for Systematic Reviews and Meta-Analyses (PRISMA) guidelines. A systematic search of PubMed, Scopus, Web of Science, Cumulative Index to Nursing and Allied Health Literature (CINAHL), and ClinicalTrials.gov was performed for randomized controlled trials (RCTs) published between January 2020 and August 2025. Eleven RCTs, encompassing 7,421 patients, were included. Data on study characteristics, efficacy outcomes, and safety outcomes were extracted. The Cochrane Risk of Bias (RoB 2) tool was used for quality assessment. JAKi monotherapy demonstrated significant efficacy, outperforming methotrexate (MTX) and placebo in American College of Rheumatology 50% (ACR50) and American College of Rheumatology 20% (ACR20) response rates, achieving high rates of disease activity score based on 28 joints with C-reactive protein (DAS28-CRP) remission, and inhibiting radiographic progression. Efficacy was comparable to tumor necrosis factor (TNF) inhibitors. Combination therapy also showed robust efficacy, often with numerical trends favoring it over monotherapy for some endpoints, though direct comparisons were limited. The safety profile was consistent across monotherapy and combination strategies, with an increased risk of infections and a low but present risk of serious adverse events (SAEs), including malignancies and cardiovascular events. JAKi monotherapy is an effective and generally well-tolerated treatment strategy for RA, offering a viable alternative for patients intolerant to csDMARDs. Its efficacy is superior to conventional DMARDs and comparable to both TNF inhibitors and JAKi combination therapy. The choice between monotherapy and combination therapy should be individualized, weighing efficacy, tolerability, and specific patient risk factors.

## Introduction and background

Rheumatoid arthritis (RA) is a chronic, systemic autoimmune disease characterized by persistent synovial inflammation, joint destruction, and progressive disability [1]. It affects approximately 0.5-1% of the global population and is associated with significant morbidity, impaired quality of life, and increased healthcare burden [2]. The primary goal of RA management is to achieve sustained disease control, prevent structural damage, and maintain long-term physical function [3].

Conventional synthetic disease-modifying antirheumatic drugs (csDMARDs), particularly methotrexate (MTX), remain the cornerstone of initial therapy for RA [4]. However, a considerable proportion of patients either fail to achieve adequate disease control or experience adverse effects that limit csDMARD use. In recent years, Janus kinase inhibitors (JAKi), an oral class of targeted synthetic DMARDs, have emerged as effective alternatives [5]. By selectively inhibiting intracellular signaling pathways implicated in RA pathogenesis, JAKi such as tofacitinib, baricitinib, upadacitinib, and filgotinib have demonstrated substantial efficacy in reducing disease activity and improving patient-reported outcomes [6].

A key question in clinical practice is whether JAKi should be used as monotherapy or in combination with csDMARDs [7]. While combination therapy with MTX has historically been favored for biologic agents due to synergistic effects and prevention of anti-drug antibody formation, JAKi, being small molecules with distinct mechanisms, may not share this limitation [8]. Some randomized controlled trials (RCTs) have suggested that JAKi monotherapy can achieve comparable clinical and radiographic outcomes to combination regimens, while others indicate a potential benefit of continued csDMARD use alongside JAK inhibition [9,10]. Moreover, the safety profiles may differ, with combination therapy potentially increasing the risk of adverse events (AEs), while monotherapy may be more tolerable but raise concerns about long-term disease control.

Despite accumulating evidence, clinical guidelines remain cautious, often recommending JAKi in combination with csDMARDs but allowing for monotherapy in patients intolerant to MTX. This variability highlights the need for a systematic synthesis of RCT data directly comparing JAKi monotherapy and combination regimens to guide evidence-based treatment decisions.

Therefore, this systematic review aims to evaluate and compare the efficacy and safety of JAKi monotherapy versus combination therapy with csDMARDs in patients with RA, drawing on evidence exclusively from RCTs.

## Review

Methodology

Protocol and Reporting

This systematic review was conducted in accordance with the Preferred Reporting Items for Systematic Reviews and Meta-Analyses (PRISMA) 2020 guidelines [11]. The protocol was developed a priori to ensure transparency in the review process, and the review methodology was structured to minimize bias and enhance reproducibility.

Eligibility Criteria

We included RCTs that directly compared JAKi monotherapy with JAKi in combination with csDMARDs in adult patients with RA. Studies published between January 2020 and August 2025 were considered eligible. The restriction to the past five years was applied to capture the most recent and clinically relevant evidence, given the rapid evolution of JAKi research and the updated safety considerations in this therapeutic area. No language restrictions were applied. Non-randomized studies, observational studies, conference abstracts without full text, reviews, editorials, and preclinical studies were excluded.

Information Sources and Search Strategy

A comprehensive literature search was conducted in the following electronic databases: PubMed, Scopus, Web of Science, Cumulative Index to Nursing and Allied Health Literature (CINAHL), and ClinicalTrials.gov. The search covered the period from January 2020 to August 2025, with the final search performed on 28 August 2025. A combination of Medical Subject Headings (MeSH) and free-text keywords was used, including terms related to “rheumatoid arthritis,” “JAK inhibitors,” “Janus kinase inhibitors,” “monotherapy,” “combination therapy,” and “csDMARDs.” Reference lists of included studies and relevant reviews were also screened to identify additional eligible trials. A full search strategy for each database is provided in Appendix 1.

Study Selection

All identified records were imported into EndNote X9 (Clarivate, London, UK) reference management software, where duplicates were removed automatically and manually checked to ensure accuracy. Titles and abstracts were independently screened by two reviewers to identify potentially eligible studies. Full texts of selected articles were retrieved and assessed for eligibility based on the predefined inclusion criteria. Disagreements were resolved through discussion and consensus, with a third reviewer consulted if necessary.

Data Extraction

Data were independently extracted by two reviewers using a standardized extraction form. Extracted data included study characteristics (author, year, country, design), patient population (sample size, demographics, disease duration), intervention details (type of JAKi, dose, duration), comparator regimen, primary and secondary efficacy outcomes (such as American College of Rheumatology 20% (ACR20), American College of Rheumatology 50% (ACR50), disease activity score based on 28 joints (DAS28), and radiographic progression), and safety outcomes (common AEs, serious AEs, and discontinuations). Any discrepancies were resolved through consensus.

Risk of Bias Assessment

The methodological quality and risk of bias of all included RCTs were assessed using the Cochrane Risk of Bias 2 (RoB 2) tool [12]. This tool evaluates bias across five domains: randomization process, deviations from intended interventions, missing outcome data, measurement of the outcome, and selection of the reported result. Each study was rated as “low risk,” “some concerns,” or “high risk” of bias. Assessments were performed independently by two reviewers, with disagreements resolved through consensus.

Data Synthesis

Given the heterogeneity of the included trials in terms of study populations, types and dosages of JAKi, comparator regimens, and outcome reporting, a meta-analysis was not performed. Pooling of results was deemed inappropriate as it could potentially obscure clinically meaningful differences and compromise the validity of conclusions. Instead, findings are presented in a structured narrative synthesis, with summary tables provided to facilitate comparison across studies.

Results

Study Selection Process

**Figure 1:**
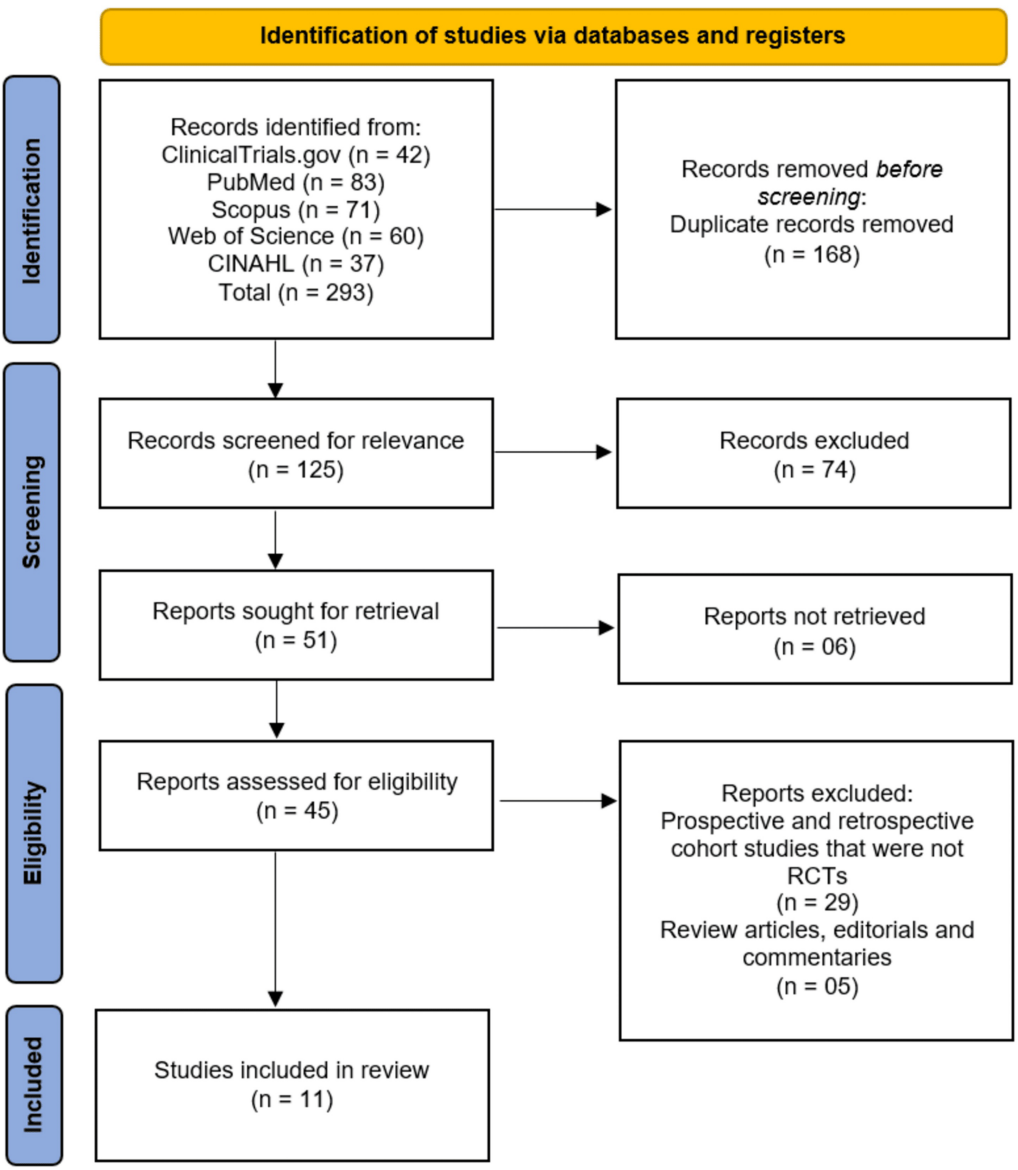
Studies' identification process on the PRISMA flowchart. PRISMA: Preferred Reporting Items for Systematic reviews and Meta-analyses; CINAHL: Cumulative Index to Nursing and Allied Health Literature; RCT: randomized controlled trials

The study selection process is detailed in the PRISMA flow diagram (Figure 1). A systematic search of electronic databases (ClinicalTrials.gov, PubMed, Scopus, Web of Science, and CINAHL) initially identified 293 records. After the removal of 168 duplicate records, 125 unique records were screened based on their titles and abstracts. Of these, 74 records were excluded for not meeting the inclusion criteria. The full texts of the remaining 51 reports were sought for retrieval, of which six could not be obtained. Subsequently, 45 full-text articles were assessed for eligibility. Upon detailed evaluation, 34 reports were excluded, with the primary reasons being that they were prospective or retrospective cohort studies that were not RCTs (n=29) or were review articles, editorials, or commentaries (n=5). This process culminated in the inclusion of 11 studies [13-23] that satisfied all eligibility criteria for this systematic review.

Study Characteristics

A total of 11 RCTs were included in this systematic review, comprising data from 7,421 patients with RA [13-23]. The characteristics of these studies are summarized in Table 1. The studies were published between 2020 and 2025, reflecting the most recent evidence on JAKi in RA. The trials were multinational or conducted in specific regions, including multicentre global studies [13,14,18,21,23], as well as those focused on populations in Japan [16,19,22], Egypt [15], and China (with additional sites in Brazil, Argentina, and South Korea) [17,20].

**Table 1:** Characteristics of included randomized controlled trials RA: rheumatoid arthritis; MTX: methotrexate; csDMARDs: conventional synthetic disease-modifying antirheumatic drugs; CDAI: Clinical Disease Activity Index; ACR: American College of Rheumatology; DAS28-CRP: disease activity score based on 28 joints with C-reactive protein; HAQ-DI: Health Assessment Questionnaire-Disability Index; TEAEs: treatment-emergent adverse events; TJC: tender joint count; SJC: swollen joint count; VAS: visual analog scale; JAKi: Janus kinase inhibitors

Author (Year)	Country	Study Design	Sample Size	Population (RA patients, prior treatment status)	Intervention (JAKi Monotherapy)	Comparator (JAKi + csDMARDs)	Follow-up Duration	Primary Outcomes Assessed
Smolen et al., [13] (2025)	Multicentre	Phase III Randomized Controlled Trial (SELECT-MONOTHERAPY, NCT02706951)	648 randomized; 598 entered long-term extension	RA patients with inadequate response to methotrexate	Upadacitinib 15 mg or 30 mg monotherapy	Methotrexate → switched to UPA15/UPA30 per protocol (csDMARD combination after week 14)	260 weeks	Safety (adverse events, exposure-adjusted event rates); Efficacy
van Vollenhoven et al., [14] (2024)	Multicentre	Phase 3, Randomized Controlled Trial, Long-Term Extension (SELECT-EARLY)	945 randomized; 775 entered LTE	Moderately to severely active RA, MTX-naïve patients	Upadacitinib 15 mg or 30 mg	MTX monotherapy; rescue therapy allowed (addition of MTX in the upadacitinib group)	5 years	Clinical response (CDAI remission, DAS28(CRP), ACR responses), structural joint progression, safety (TEAEs, serious AEs)
Mahmoud et al., [15] (2024)	Egypt	Randomized Controlled Rrial	334	RA patients, refractory to cDMARDs	Baricitinib	TNF-α inhibitors, cDMARDs	24 weeks	TJC, SJC, VAS, DAS28, CDAI, HAQ-DI, Larsen score, ACR 20/50/70 response
Harigai et al., [16] (2020)	Japan	Integrated analysis of 6 studies (5 Phase 2/3 RCTs + 1 long-term extension)	514 RA patients (851.5 patient-years exposure)	Moderately to severely active RA	Baricitinib monotherapy (various doses)	The focus was on pooled baricitinib exposure	Median 1.7 years (max 3.2 years)	Safety outcomes
Li et al., [17] (2020)	China, Brazil, Argentina (majority from China)	Phase 3, double-blind, randomized, placebo-controlled trial	290	Patients with moderately to severely active RA and inadequate response to MTX	Baricitinib 4 mg once daily	Placebo (patients continued background MTX)	52 weeks (primary analysis at 12 weeks)	ACR20 response at week 12; secondary outcomes included HAQ-DI, DAS28
van Vollenhoven et al., [18] (2020)	Multinational (43 countries)	Phase 3, randomized, controlled trial	947	Predominantly early RA patients, MTX-naïve or with limited MTX exposure	Upadacitinib 15 mg once daily OR Upadacitinib 30 mg once daily (monotherapy)	Methotrexate 7.5–20 mg/week	24 weeks	ACR50 response at week 12; DAS28-CRP <2.6 at week 24; radiographic progression; PROs
Kameda et al., [19] (2020)	Japan	Multicentre, Phase IIb/III, randomized, double-blind, dose-ranging RCT	197 (187 completed)	Adults with active RA and inadequate response to csDMARDs (on previously stable csDMARDs)	Upadacitinib monotherapy (7.5 mg, 15 mg, 30 mg once daily)	Placebo + background csDMARDs	12 weeks	ACR20 response at week 12 (primary); ACR50, ACR70, DAS28-CRP remission/low disease activity, safety outcomes (secondary)
Zeng et al., [20] (2021)	China, Brazil, South Korea	Randomized, double-blind, placebo-controlled	338	Patients with active RA and inadequate response (IR) to csDMARDs	Upadacitinib 15 mg once daily	Placebo + stable csDMARDs	12 weeks	ACR20 at week 12
Westhovens et al., [21] (2021)	Multicentre	Phase 3, multicentre, double-blind RCT	1252	Patients with active RA, limited or no prior MTX exposure	Filgotinib 200 mg daily (FIL200)	Filgotinib 200 mg + MTX (FIL200+MTX), Filgotinib 100 mg + MTX (FIL100+MTX)	52 weeks	ACR20 response at week 24, DAS28(CRP) remission, HAQ-DI changes
Atsumi et al., [22] (2023)	Japan	Randomized controlled trial (subanalysis of global RCT)	56	Japanese RA patients with limited or no prior methotrexate exposure	Filgotinib 200 mg	Filgotinib 200 mg + MTX, Filgotinib 100 mg + MTX	52 weeks (main RCT) + LTE to 48 weeks	Efficacy (ACR20) and Safety (adverse events)
Combe et al., [23] (2021)	Multicentre	Phase III, multicentre, double-blind, placebo-controlled, and active-controlled RCT	1755	Patients with active RA despite ongoing MTX treatment	Filgotinib 200 mg or 100 mg once daily	Adalimumab 40 mg biweekly + stable MTX; placebo + stable MTX	52 weeks (placebo-controlled through week 24)	ACR20 at week 12; additional efficacy outcomes including RA signs and symptoms, physical function, structural damage; safety

The sample sizes of the included trials varied, ranging from 56 [22] to 1,755 [23] randomized patients. The studied populations encompassed a broad spectrum of RA disease activity and prior treatment exposure, including MTX-naïve patients [14,18,21,22], those with an inadequate response to MTX [13,17,23] or csDMARDs [15,19,20], and patients with limited prior MTX exposure [21,22]. The JAKi evaluated were upadacitinib (15 mg or 30 mg once daily) [13,14,18,20], baricitinib (2 mg or 4 mg once daily) [15,17], and filgotinib (100 mg or 200 mg once daily) [21-23].

The design of the interventions was a key focus of this review. Six studies specifically investigated JAKi monotherapy [13,14,16,18,19,21], while five studies included arms where JAKi was used in combination with a csDMARD, primarily MTX [17,21,23]. One study featured a design where the monotherapy group could have csDMARDs added after week 14 as rescue therapy [14], and another directly compared a JAKi (baricitinib) to both TNF inhibitors and conventional DMARDs [3]. The follow-up duration across the studies varied from 12 weeks [19,20] to 260 weeks (five years) [13,14], allowing for the assessment of both short-term efficacy and long-term safety and durability.

Efficacy outcomes

Clinical Response Measures

JAKi monotherapy demonstrated superior efficacy compared to placebo and active comparators across multiple measures of clinical response. In MTX-naïve patients, upadacitinib monotherapy (15 mg and 30 mg) showed significantly higher ACR50 response rates at week 12 (52% and 56%, respectively) compared to MTX monotherapy (28%) [18]. Similarly, in patients with an inadequate response to csDMARDs, upadacitinib monotherapy at 15 mg and 30 mg doses yielded ACR20 response rates of 71.6% [20] and 83.7% [19], significantly outperforming placebo. Baricitinib 4 mg monotherapy also showed a significantly higher ACR20 response rate (58.6%) compared to placebo (28.3%) in MTX inadequate responders at week 12 [17]. In a direct comparative study, baricitinib monotherapy demonstrated higher ACR20/50/70 response rates at week 12 compared to conventional DMARDs and was comparable to tumor necrosis factor (TNF) inhibitors [15].

Disease Activity and Remission Rates

JAKi monotherapy was consistently effective in reducing disease activity and achieving remission. A high proportion of patients on upadacitinib monotherapy achieved a DAS28-C-reactive protein (CRP) of less than 2.6 (remission) at week 24 (48% and 50% for 15 mg and 30 mg, respectively, versus 19% for MTX) [18]. Long-term data from the SELECT-MONOTHERAPY study at 260 weeks showed that approximately 42% of patients on upadacitinib monotherapy maintained a Clinical Disease Activity Index (CDAI) score of ≤10 and a DAS28-CRP of ≤3.2 [13]. Filgotinib monotherapy (200 mg) demonstrated DAS28-CRP remission rates of 43-54% [21].

Functional and Radiographic Outcomes

Improvements in physical function, as measured by the Health Assessment Questionnaire-Disability Index (HAQ-DI), were observed with JAKi treatment. Baricitinib 4 mg monotherapy showed a significant improvement in HAQ-DI score compared to placebo [17]. In the study by Mahmoud et al., baricitinib improved functional status more than conventional DMARDs and was comparable to TNF inhibitors [15]. Furthermore, JAKi monotherapy showed efficacy in inhibiting structural joint damage. Upadacitinib monotherapy resulted in a greater inhibition of radiographic progression compared to MTX over five years, with 88-89% of patients showing no progression versus 78% on MTX [14,18]. Baricitinib also delayed radiographic progression more effectively than conventional DMARDs [15].

Combination Therapy vs. Monotherapy

The efficacy of JAKi in combination with csDMARDs was also robust. Filgotinib 200 mg in combination with MTX achieved an ACR20 response of 76.6% at week 12, demonstrating non-inferiority to adalimumab combined with MTX [23]. Another trial found that filgotinib 200 mg combined with MTX or as monotherapy yielded ACR20 responses of 78-81% [21]. However, direct head-to-head comparisons between JAKi monotherapy and JAKi + csDMARD combination therapy within the same study were limited. The available data, such as from the FINCH 3 trial, suggest that filgotinib combination therapy and monotherapy arms both performed favorably against MTX alone, but numerical trends often favored the combination therapy arm for certain endpoints [21].

Safety outcomes

The safety profile of JAKi, both as monotherapy and in combination with csDMARDs, was evaluated across all studies, with the key outcomes detailed in Table 2. The most commonly reported AEs across all JAKi classes included infections, with herpes zoster being a notable concern.

**Table 2:** Efficacy and safety outcomes of JAK inhibitors as monotherapy vs. combination therapy AEs: adverse events; NR: not reported; RA: rheumatoid arthritis; MTX: methotrexate; csDMARDs: conventional synthetic disease-modifying antirheumatic drugs; CDAI: Clinical Disease Activity Index; ACR: American College of Rheumatology; DAS28-CRP: disease activity score based on 28 joints with C-reactive protein; HAQ-DI: Health Assessment Questionnaire-Disability Index; TEAEs: treatment-emergent adverse events; JAKi: Janus kinase inhibitors; TNF: tumor necrosis factor; GIT: gastrointestinal tract; CVS: cardiovascular system; LTE: long-term extension; CK: creatine kinase; DVT: deep vein thrombosis; VTE: venous thromboembolism

Author (Year)	JAK Inhibitor Used	csDMARD Used (if applicable)	ACR20/50/70 Response Rates (%)	DAS28-CRP/ESR Improvement	Radiographic Progression	HAQ-DI Change	Adverse Events (Any, Serious, Discontinuations)
Smolen et al., [13] (2025)	Upadacitinib 15/30 mg	None (Monotherapy)	NR	CDAI ≤10: ~42%; DAS28-CRP ≤3.2: ~42% at week 260	NR	NR	Discontinuations 14.5%; AEs: herpes zoster, non-melanoma skin cancer, hepatic disorder, neutropenia, lymphopenia, CK elevation; Serious AEs NR
van Vollenhoven et al., [14] (2024)	Upadacitinib 15/30 mg	None	Improved vs. MTX	Better DAS28(CRP) vs. MTX	Greater inhibition vs. MTX	NR	TEAEs were higher with 30 mg; 15 mg was favorable; serious AEs and discontinuations were higher with 30 mg
Mahmoud et al., [15] (2024)	Baricitinib vs. TNF inhibitors vs. cDMARDs	Baricitinib: None; TNF: None; cDMARDs: —	Baricitinib showed higher ACR20/50/70 vs. cDMARDs; ACR70 at week 12 higher vs. TNF inhibitors; TNF comparable; cDMARDs lower	Baricitinib and TNF improved; cDMARDs showed less improvement	Baricitinib delayed progression more than cDMARDs; TNF not specified	Baricitinib improved functional status; TNF comparable; cDMARDs less	Baricitinib: infection, GIT, CVS; TNF: infection, skin; cDMARDs: mostly GIT; overall comparable safety between baricitinib and TNF inhibitors
Harigai et al., [16] (2020)	Baricitinib (JAK1/JAK2)	NR	NR	NR	NR	NR	Any AEs: EAIR 57.4/100PY; serious infections: 3.6/100PY; herpes zoster: 6.5/100PY (more frequent in Japanese patients); malignancies: 1.1/100PY (including 2 lymphomas); major CV AEs: 0.3/100PY; GI perforation: 0.1/100PY; DVT: 0.5/100PY; no deaths reported
Li et al., [17] (2020)	Baricitinib (4 mg daily)	Background MTX (patients were MTX inadequate responders)	ACR20: 58.6% vs. 28.3% placebo at week 12; ACR50/70 not reported	Significant improvement in DAS28-hsCRP (vs. placebo)	NR	Significant improvement vs. placebo	Any AEs: higher with baricitinib vs. placebo; serious AEs: similar to placebo; discontinuations: not reported
van Vollenhoven et al., [18] (2020)	Upadacitinib 15 mg and 30 mg (monotherapy)	None	ACR50: 52% (15 mg), 56% (30 mg) vs. MTX 28%; ACR20/70 not reported	DAS28-CRP <2.6: 48% (15 mg), 50% (30 mg) vs. MTX 19%	No progression: 88–89% vs. MTX 78%	Improved	Any AE: 64% (15 mg), 71% (30 mg) vs. MTX 65%; deaths: 2 (15 mg), 3 (30 mg), 1 (MTX)
Kameda et al., [19] (2020)	Upadacitinib (7.5, 15, 30 mg QD)	Stable csDMARDs	ACR20: 75.5% (7.5 mg), 83.7% (15 mg), 80.0% (30 mg) vs. 42.9% placebo; ACR50/70 also significantly higher vs. placebo	Higher DAS28-CRP remission (<2.6) and low disease activity rates with upadacitinib vs. placebo; numerically better with 15 and 30 mg than 7.5 mg	NR	NR	Adverse events and infections more common with upadacitinib vs. placebo; numerically highest at 30 mg. Serious infections, opportunistic infections, herpes zoster reported; no venous thromboembolic events.
Zeng et al., [20] (2021)	Upadacitinib 15 mg	Yes (background csDMARDs)	ACR20: 71.6%, ACR50: NR, ACR70: NR	DAS28-CRP <2.6 achieved	NR	NR	Serious infections: 2.4%, herpes zoster: 1.8%, VTE: 1 case, discontinuations: NR
Westhovens et al., [21] (2021)	Filgotinib 200/100 mg	Methotrexate (combo) / None (monotherapy)	ACR20: 78–81% (ACR50/70 not reported)	DAS28-CRP <2.6: 43–54% (monotherapy not reported)	NR	−0.94 to −1.0	Comparable to MTX; numbers not reported
Atsumi et al., [22] (2023)	Filgotinib 200 mg / 100 mg	± MTX	ACR20: 75–83%; ACR50/70: NR	NR	NR	NR	Comparable across groups at Week 52 and LTE to Week 48
Combe et al., [23] (2021)	Filgotinib 200 and 100 mg	MTX	ACR20: 76.6% (FIL200), 69.8% (FIL100); ACR50/70: NR	FIL200 non-inferior to adalimumab; FIL100 not non-inferior	Inhibited	Improved	Comparable to adalimumab; NR

*Infections * 

Rates of any AEs and infections were generally higher with JAKi treatment compared to placebo or MTX. The incidence of herpes zoster was elevated with JAKi use, particularly in Asian populations; an integrated analysis of baricitinib trials in Japanese patients reported an exposure-adjusted incidence rate (EAIR) of 6.5 events per 100 patient-years [16]. Serious infection rates were reported to be between 3.6 per 100 patient-years for baricitinib [16] and 2.4% for upadacitinib over 12 weeks [20].

Serious Adverse Events and Malignancy

The incidence of serious AEs (SAEs) was variable across studies. In the long-term extension of the SELECT-EARLY trial, the rate of SAEs and discontinuations due to AEs was higher with upadacitinib 30 mg compared to the 15 mg dose [14]. Malignancies were reported at an EAIR of 1.1 per 100 patient-years for baricitinib, which included cases of lymphoma [16].

Cardiovascular and Thrombotic Risk

Major adverse cardiovascular events (MACE) and venous thromboembolism (VTE) were monitored closely. In the Japanese baricitinib analysis, the EAIR for major cardiovascular events was 0.3 per 100 patient-years, and for deep vein thrombosis (DVT) was 0.5 per 100 patient-years [16]. Most studies, such as the one by Kameda et al., reported no VTE events [19], though one case of VTE was reported in a study of upadacitinib [20].

Comparative Safety

The safety profile of JAKi monotherapy appeared generally consistent with that of combination therapy, with the addition of csDMARDs potentially contributing to an increased burden of AEs like gastrointestinal intolerance. In the study comparing baricitinib to TNF inhibitors and conventional DMARDs, the overall safety was reported to be comparable between baricitinib and TNF inhibitors [15]. Similarly, the safety of filgotinib was comparable to both MTX and adalimumab [21,23].

Risk of bias assessment

The methodological quality of the included studies, as assessed by the Cochrane ROB 2 tool, was generally high, though one study was deemed to be at high risk of bias. The majority of trials [13,14,16-21,23] were judged to be at low risk of bias overall, demonstrating robust methods in random sequence generation, allocation concealment, low levels of missing outcome data, appropriate blinding, and pre-specified analysis plans. One study [22] raised some concerns, primarily due to potential deviations from intended interventions in its long-term extension phase. In contrast, the study by Mahmoud et al. [15] was assessed as having a high overall risk of bias. This judgment was based on some concerns in the randomization process and selection of reported results, a high risk of bias due to deviations from intended interventions (as it was a non-blinded study), and a high risk of bias in the measurement of outcomes due to the lack of blinding of participants and personnel. Consequently, while the findings from most studies are considered reliable, the results from [15] must be interpreted with greater caution (Table 3).

**Table 3:** Assessment of risk of bias on the Cochrane RoB 2 tool

Study (Author, Year)	Randomization Process	Deviations from Intended Interventions	Missing Outcome Data	Measurement of the Outcome	Selection of Reported Result	Overall Bias
Smolen et al., [13] (2025)	Low	Low	Low	Low	Low	Low
van Vollenhoven et al., [14] (2024)	Low	Low	Low	Low	Low	Low
Mahmoud et al., [15] (2024)	Some concerns	High	Some concerns	High	Some concerns	High
Harigai et al., [16] (2020)	Low	Low	Low	Low	Low	Low
Li et al., [17] (2020)	Low	Low	Low	Low	Low	Low
van Vollenhoven et al., [18] (2020)	Low	Low	Low	Low	Low	Low
Kameda et al., [19] (2020)	Low	Low	Low	Low	Low	Low
Zeng et al., [20] (2021)	Low	Low	Low	Low	Low	Low
Westhovens et al., [21] (2021)	Low	Low	Low	Low	Low	Low
Atsumi et al., [22] (2023)	Low	Some concerns	Low	Low	Low	Some concerns
Combe et al., [23] (2021)	Low	Low	Low	Low	Low	Low

Discussion

This systematic review comprehensively evaluated the efficacy and safety of JAKi monotherapy versus combination therapy with csDMARDs in patients with RA by synthesizing evidence from 11 RCTs [13-23]. The findings indicate that JAKi monotherapy, including upadacitinib, baricitinib, and filgotinib, demonstrates robust efficacy across multiple clinical, functional, and radiographic outcomes, often superior to conventional DMARDs or placebo and comparable to TNF inhibitors. Specifically, JAKi monotherapy resulted in significantly higher ACR response rates, improved disease activity scores, greater achievement of remission, enhanced physical function, and inhibition of radiographic progression compared to MTX or placebo in both MTX-naïve and MTX-inadequate responder populations [13,14,17-19]. For instance, upadacitinib monotherapy at doses of 15 mg and 30 mg yielded ACR50 responses of 52% and 56%, respectively, in MTX-naïve patients, significantly outperforming MTX monotherapy (28%) [18]. Similarly, baricitinib 4 mg monotherapy showed superior ACR20 responses compared to placebo in MTX-inadequate responders [17]. These results align with prior studies such as the ORAL Solo trial, which reported that tofacitinib monotherapy was significantly more effective than placebo in patients with an inadequate response to DMARDs [24]. Furthermore, the sustained efficacy observed in long-term extensions, such as the SELECT-MONOTHERAPY study, where approximately 42% of patients maintained low disease activity after 260 weeks of upadacitinib monotherapy [13], reinforces the durability of response, which is consistent with findings from the long-term extension of the tofacitinib RA program [25].

When comparing JAKi monotherapy to combination therapy, the current evidence suggests that both strategies are effective, though direct head-to-head comparisons within the same trial are limited. In studies where JAKi was used in combination with MTX, such as with filgotinib, high ACR20 response rates (76.6%) were achieved, demonstrating non-inferiority to adalimumab combined with MTX [23]. However, numerical trends in some trials, like FINCH 3, often favored combination therapy over monotherapy for certain endpoints, though both were superior to MTX alone [21]. This is reminiscent of findings from a study where tofacitinib combination therapy showed marginally better efficacy than monotherapy in MTX-inadequate responders, though the differences were not always statistically significant [26]. The comparable efficacy between monotherapy and combination therapy is particularly noteworthy as it offers a valuable treatment strategy for patients who are intolerant to MTX or other csDMARDs, thereby expanding therapeutic options. This is especially relevant in real-world settings where csDMARD intolerance is common, and monotherapy with a highly effective agent like a JAKi could improve adherence and patient satisfaction.

The safety profile of JAKi, both as monotherapy and in combination with csDMARDs, was consistent with known class effects, characterized by an increased risk of infections, particularly herpes zoster, and a potential for SAEs such as MACE, VTE, and malignancies. Herpes zoster incidence was notably higher in Asian populations, as evidenced by an EAIR of 6.5 events per 100 patient-years in Japanese patients treated with baricitinib [16], which is consistent with prior observations of elevated herpes zoster risk in Asian RA patients receiving JAKi, as reported in a meta-analysis by Cohen et al. [27]. The risk of serious infections was generally low but present, with rates ranging from 2.4% over 12 weeks for upadacitinib [20] to 3.6 per 100 patient-years for baricitinib [16]. These findings are in line with the integrated safety analysis of tofacitinib, which showed a similar range of serious infection events [28]. Additionally, the incidence of malignancies, including lymphoma, was reported at an EAIR of 1.1 per 100 patient-years for baricitinib [16], which is consistent with the known slightly elevated malignancy risk associated with JAKi, as highlighted in a recent network meta-analysis by Xie et al. [29]. Cardiovascular and thrombotic risks, though monitored closely, were relatively low, with major cardiovascular events reported at an EAIR of 0.3 per 100 patient-years and DVT at 0.5 per 100 patient-years for baricitinib [16]. Most studies did not report significant VTE events, though one case was noted in an upadacitinib study [20]. This aligns with the ongoing discourse around JAKi safety, particularly following the ORAL Surveillance study, which showed an increased risk of MACE and malignancy with tofacitinib compared to TNF inhibitors in high-risk populations [30]. However, it is important to note that the patients in the included studies of this review may not fully represent this high-risk population, and the overall cardiovascular and thrombotic risks observed here were modest.

The safety profile of JAKi monotherapy appeared generally consistent with that of combination therapy, though the addition of csDMARDs may contribute to an increased burden of AEs, such as gastrointestinal intolerance. In the study by Mahmoud et al., the overall safety was comparable between baricitinib monotherapy and TNF inhibitors, though the high risk of bias in this study necessitates cautious interpretation [15]. Similarly, the safety of filgotinib was comparable to both MTX and adalimumab [21,23]. These findings suggest that the addition of csDMARDs may not significantly alter the safety profile of JAKi beyond the known risks associated with each individual drug, but it may introduce additional AEs related to csDMARDs, such as hepatotoxicity or cytopenias. This is consistent with a systematic review by Lee et al., which found that the safety profiles of JAKi were largely similar whether used as monotherapy or in combination with csDMARDs, though combination therapy had a slightly higher rate of discontinuation due to AEs [31].

When comparing the efficacy and safety of JAKi to other biologic DMARDs, the findings of this review suggest that JAKi are at least non-inferior to TNF inhibitors in terms of efficacy, with a similar safety profile, though with a different risk burden. For example, baricitinib showed comparable efficacy to TNF inhibitors in the study by Mahmoud et al. [15], and filgotinib was non-inferior to adalimumab when both were combined with MTX [23]. This aligns with network meta-analyses, such as the one by Singh et al., which found that JAKi have similar efficacy to TNF inhibitors but with a different safety profile, particularly regarding herpes zoster and thrombotic risk [32]. However, the recent ORAL Surveillance study has raised concerns about the cardiovascular and malignancy risks of JAKi compared to TNF inhibitors in older patients with cardiovascular risk factors [30], highlighting the importance of patient selection and risk stratification when choosing between these agents.

The findings of this review have several clinical implications. First, JAKi monotherapy represents an effective and viable option for patients who are intolerant to csDMARDs or who prefer monotherapy for reasons of adherence or convenience. The robust efficacy and acceptable safety profile make it a valuable alternative to combination therapy, particularly in settings where csDMARD use is limited by toxicity or contraindications. Second, the comparable efficacy between monotherapy and combination therapy suggests that for some patients, monotherapy may be sufficient to achieve disease control, thereby avoiding the cumulative toxicity associated with csDMARDs. However, the numerical trends favoring combination therapy in some studies indicate that for certain patients, particularly those with high disease activity or poor prognostic factors, combination therapy may still be preferable. Finally, the safety findings underscore the need for vigilant monitoring for infections, herpes zoster, cardiovascular events, and malignancies in patients receiving JAKi, regardless of whether they are used as monotherapy or in combination with csDMARDs.

This systematic review has several limitations. First, the included studies varied in design, patient populations, follow-up duration, and outcome measures, which may introduce heterogeneity and limit the comparability of results. For example, some studies focused on MTX-naïve patients [14,18,21,22], while others included patients with inadequate response to MTX or other csDMARDs [13,15,17,19,20,23]. Second, the majority of studies were industry-sponsored, which may introduce potential bias in the reporting of efficacy and safety outcomes. Third, direct head-to-head comparisons between JAKi monotherapy and JAKi combination therapy were limited, making it difficult to draw definitive conclusions about the superiority of one strategy over the other. Most comparisons were indirect, against placebo or active comparators like MTX or TNF inhibitors. Fourth, the safety data, particularly for rare events like malignancies and cardiovascular events, were based on relatively short-term follow-up in some studies, and longer-term data are needed to fully characterize the risk profile. Fifth, one study had a high risk of bias due to its non-blinded design [15], which may affect the reliability of its findings. Finally, the generalizability of the findings may be limited by the underrepresentation of certain patient populations, such as those with high cardiovascular risk or elderly patients, who were excluded from many of the trials.

## Conclusions

JAKi monotherapy is an effective and generally well-tolerated treatment strategy for patients with RA, showing superior efficacy to conventional DMARDs and placebo and comparable efficacy to TNF inhibitors and JAKi combination therapy. The safety profile is consistent with known class effects, including an increased risk of infections, herpes zoster, and potentially SAEs such as cardiovascular events and malignancies, though the absolute risks remain relatively low. The choice between monotherapy and combination therapy should be individualized based on patient preferences, tolerance to csDMARDs, and risk factors for AEs. Future research should focus on direct comparisons between JAKi monotherapy and combination therapy, long-term safety assessments, and the identification of biomarkers to predict response and toxicity, thereby enabling more personalized treatment approaches.

## Appendices

Appendix 1 

**Table 4:** Search strategy

Database	Search String
PubMed	("Rheumatoid Arthritis"[Mesh] OR "rheumatoid arthritis"[tiab]) AND ("Janus Kinase Inhibitors"[Mesh] OR "JAK inhibitor*"[tiab] OR "tofacitinib"[tiab] OR "baricitinib"[tiab] OR "upadacitinib"[tiab] OR "filgotinib"[tiab]) AND ("Antirheumatic Agents"[Mesh] OR "conventional synthetic DMARD*"[tiab] OR "csDMARD*"[tiab] OR "methotrexate"[tiab] OR "leflunomide"[tiab] OR "sulfasalazine"[tiab] OR "hydroxychloroquine"[tiab]) AND ("Monotherapy"[tiab] OR "Combination Therapy"[tiab]) AND ("Randomized Controlled Trial"[Publication Type] OR "randomized"[tiab] OR "placebo-controlled"[tiab])
Scopus	(TITLE-ABS-KEY("rheumatoid arthritis") AND TITLE-ABS-KEY("JAK inhibitor*" OR tofacitinib OR baricitinib OR upadacitinib OR filgotinib) AND TITLE-ABS-KEY("csDMARD*" OR "methotrexate" OR "leflunomide" OR "sulfasalazine" OR "hydroxychloroquine") AND TITLE-ABS-KEY("monotherapy" OR "combination therapy") AND TITLE-ABS-KEY("randomized" OR "randomised" OR "placebo-controlled"))
Web of Science	TS=("rheumatoid arthritis") AND TS=("JAK inhibitor*" OR tofacitinib OR baricitinib OR upadacitinib OR filgotinib) AND TS=("csDMARD*" OR methotrexate OR leflunomide OR sulfasalazine OR hydroxychloroquine) AND TS=("monotherapy" OR "combination therapy") AND TS=("randomized" OR "randomised" OR "placebo-controlled")
CINAHL (via EBSCOhost)	("rheumatoid arthritis" AND ("JAK inhibitor*" OR tofacitinib OR baricitinib OR upadacitinib OR filgotinib) AND ("csDMARD*" OR methotrexate OR leflunomide OR sulfasalazine OR hydroxychloroquine) AND ("monotherapy" OR "combination therapy") AND ("randomized controlled trial" OR "clinical trial"))
ClinicalTrials.gov	Condition or disease: Rheumatoid Arthritis AND Other terms: ("JAK inhibitor" OR tofacitinib OR baricitinib OR upadacitinib OR filgotinib) AND ("monotherapy" OR "combination therapy" OR "methotrexate")
